# Augmented Renal Clearance in Severe Infections—An Important Consideration in Vancomycin Dosing: A Narrative Review

**DOI:** 10.3389/fphar.2022.835557

**Published:** 2022-03-21

**Authors:** Qile Xiao, Hainan Zhang, Xiaomei Wu, Jian Qu, Lixia Qin, Chunyu Wang

**Affiliations:** ^1^ Department of Neurology, Second Xiangya Hospital, Central South University, Changsha, China; ^2^ Department of Pharmacy, Second Xiangya Hospital, Central South University, Changsha, China

**Keywords:** vancomycin, critically ill, infection, augmented renal clearance, pharmacokinetics/pharmacodynamics

## Abstract

Vancomycin is a hydrophilic antibiotic widely used in severe infections, including bacteremia and central nervous system (CNS) infections caused by Gram-positive bacteria such as methicillin-resistant *Staphylococcus aureus* (MRSA), coagulase-negative staphylococci and enterococci. Appropriate antimicrobial dosage regimens can help achieve the target exposure and improve clinical outcomes. However, vancomycin exposure in serum and cerebrospinal fluid (CSF) is challenging to predict due to rapidly changing pathophysiological processes and patient-specific factors. Vancomycin concentrations may be decreased for peripheral infections due to augmented renal clearance (ARC) and increased distribution caused by systemic inflammatory response syndrome (SIRS), increased capillary permeability, and aggressive fluid resuscitation. Additionally, few studies on vancomycin’s pharmacokinetics (PK) in CSF for CNS infections. The relationship between exposure and clinical response is unclear, challenging for adequate antimicrobial therapy. Accurate prediction of vancomycin pharmacokinetics/pharmacodynamics (PK/PD) in patients with high interindividual variation is critical to increase the likelihood of achieving therapeutic targets. In this review, we describe the interaction between ARC and vancomycin PK/PD, patient-specific factors that influence the achievement of target exposure, and recent advances in optimizing vancomycin dosing schedules for severe infective patients with ARC.

## Introduction

Vancomycin is the first-line antibiotic for treating methicillin-resistant *Staphylococcus aureus* (MRSA) infection and other Gram-positive cocci infections. 90% of vancomycin is cleared by the prototype through the kidney. As a result, renal function significantly impacts on its pharmacokinetics/pharmacodynamics (PK/PD) (Rybak et al., 2020). For severe infections, augmented renal clearance (ARC), which refers to the increased renal elimination of circulating solutes, is common. It can be caused by the disease itself, the inflammatory state, or therapeutic interventions (Bilbao-Meseguer et al., 2018). Current studies suggest that ARC can lead to increased vancomycin clearance (CL_V_), resulting in subtherapeutic serum concentrations, which increases the risk of treatment failure and bacterial drug resistance (Rybak et al., 2020). In addition, severely altered and variable PK affected by patient-specific factors could also result in subexposure (Landersdorfer and Nation, 2021). Higher doses more than recommended by current guidelines are needed to attain the desired drug exposure in these patients (Hobbs et al., 2015; Mahmoud and Shen, 2017; Udy et al., 2018). Furthermore, for central nervous system (CNS) infections, vancomycin enters the cerebrospinal fluid (CSF) *via* paracellular pathways. Its permeability to CSF is limited under normal conditions due to its high molecular weight and hydrophilicity (Kumta et al., 2018). The blood-brain barrier (BBB) and the blood-cerebrospinal fluid barrier (BCB) can be disrupted by meningitis or neurosurgery, resulting in increased vancomycin permeability, which varies greatly between individuals (Beach et al., 2017; Tiede et al., 2021). The drug exposure target at the infection site such as CSF and the protocol required to achieve the target remain unclear. Optimizing antibiotic therapy is essential to increase targeted exposure ratios and improve clinical outcomes (Landersdorfer and Nation, 2021). However, only a few studies have investigated the effect of ARC on vancomycin PK/PD, and little is known about its PK for CNS infections like meningitis or ventriculitis. The purpose of this review is to summarize the pathophysiological mechanism of ARC, the effects of ARC and patient-specific factors on vancomycin PK/PD, and the progress of optimal delivery of vancomycin for severe infective patients with ARC.

## Definition, Epidemiology and Risk Factors of Augmented Renal Clearance

ARC was first described by Udy AA et al. and defined as enhanced elimination of solutes compared to baseline, a process involving altered glomerular filtration and tubular function (Udy et al., 2010a). Accurately defining the process of enhanced renal clearance is challenging and depends on what is accepted as “normal” renal function in a particular population (Udy et al., 2010b). For instance, young people’s normal glomerular filtration rate (GFR) is about 125 ml/min/1.73 m^2^, but these values decrease with age (Kidney Disease: Improving Global Outcomes (KDIGO) CKD Work Group, 2013). Therefore, data describing the optimal diagnostic thresholds and corresponding cut-off value for increased creatinine clearance in a particular population are lacking. Most subsequent studies have used creatinine clearance (CrCl) ≥130 ml/min/1.73 m^2^ as the diagnostic criterion for ARC, since there are some clinical or PK studies that have linked CrCl >130 ml/min/1.73 m^2^ to antimicrobial subexposure (Udy et al., 2013; Udy et al., 2014; Huttner et al., 2015), although some studies have defined lower (≥120 ml/min/1.73 m^2^) (Campassi et al., 2014; May et al., 2015) or higher thresholds (≥150 ml/min/1.73 m^2^) (Carrié et al., 2019).

ARC was found to be present in 4.1–65.1% of critically ill patients (Udy et al., 2014; De Waele et al., 2015; Ruiz et al., 2015; Hirai et al., 2016; Tsai et al., 2018; Nei et al., 2020; Beunders et al., 2021), and more common in certain subgroups such as trauma or multiple injuries (57.0–85.7%) (Udy et al., 2013; Barletta et al., 2016; Barletta et al., 2017; Mulder et al., 2019). Moreover, observational studies have found that the incidence of ARC is higher in neurological diseases like traumatic brain injury (TBI) (Dias et al., 2015; Udy et al., 2017a), intracerebral hemorrhage (ICH) (Morbitzer et al., 2019), and subarachnoid hemorrhage (SAH), which are 83.0–88.0%, 50.0%, and 94.0–100.0%, respectively. For CNS infections, ARC has an incidence of 25.0–47.0% in patients with bacterial meningitis (BM), ventriculitis, or neurosurgery (Lautrette et al., 2012; Blassmann et al., 2019; Chen et al., 2020). ARC patients are more likely to be younger (De Waele et al., 2015), and it can occur transiently or persist during hospitalization (Udy et al., 2014; De Waele et al., 2015). Renal clearance may increase over time (Udy et al., 2014; Morbitzer et al., 2019), suggesting the need for dynamic monitoring of renal function for drug dose adjustment.

**FIGURE 1:**
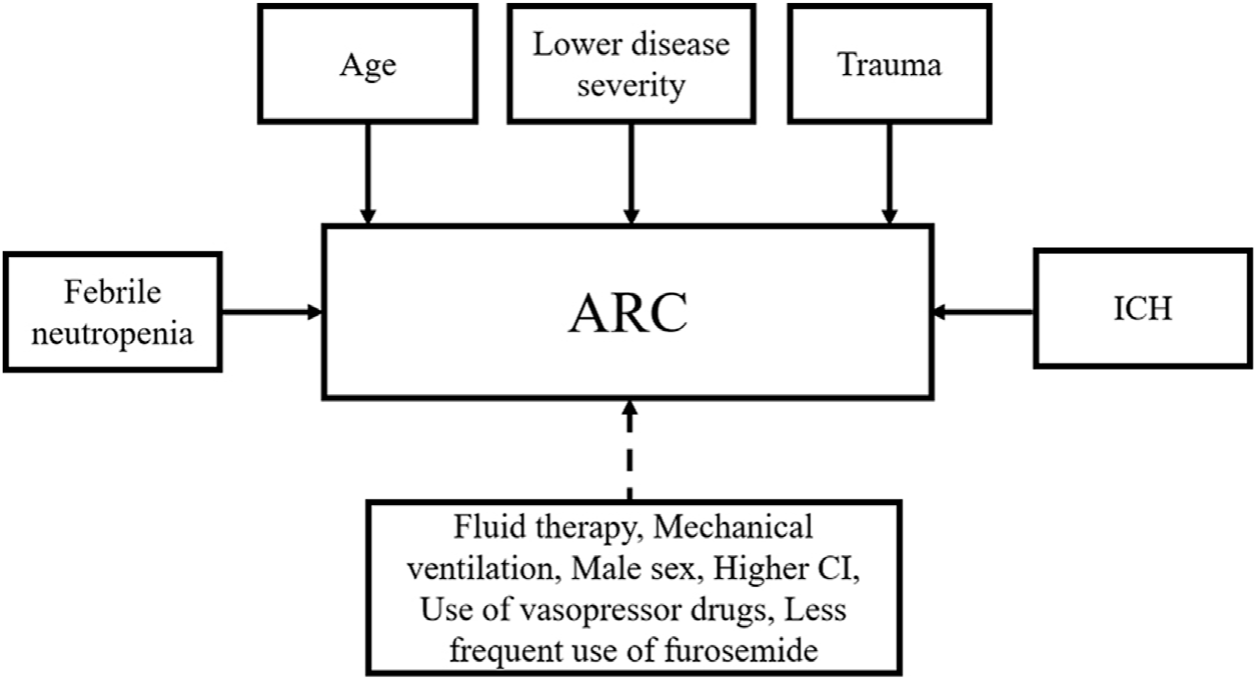
Risk factors associated with ARC. ARC, augmented renal clearance; ICH, intracerebral hemorrhage; CI, cardiac index. Solid lines represent identified factors and dotted lines represent undetermined factors.

ARC may occur due to a variety of factors (Figure 1). Younger age has been a significant predictor of ARC in univariate and multivariate analyses (Kawano et al., 2016; Barletta et al., 2017; Mulder et al., 2019; Nei et al., 2020; Nazer et al., 2021). Some studies have also found that lower disease severity (Udy et al., 2013; Huttner et al., 2015; Kawano et al., 2016; Udy et al., 2017b; Nei et al., 2020), trauma (Udy et al., 2013; Campassi et al., 2014; Huttner et al., 2015; Ruiz et al., 2015; Nei et al., 2020), and ICH (Nei et al., 2020) are independent risk factors associated with ARC. Besides, febrile neutropenia was investigated to be an independent risk factor for ARC in critically ill patients (OR 5.86, 95% CI 1.98 ± 66, *p* = 0.0030) (Hirai et al., 2016), but there is a lack of further studies to confirm this result. Other risk factors that may promote the development of ARC include male sex (Udy et al., 2013; Barletta et al., 2017; Mulder et al., 2019), fluid therapy (Mulder et al., 2019; Dhondt et al., 2020), mechanical ventilation (Udy et al., 2014; Nazer et al., 2021), higher cardiac index (CI) (Udy et al., 2013; Barletta et al., 2016), use of vasopressor drugs (Udy et al., 2010c; Nazer et al., 2021), and less frequent use of furosemide (Campassi et al., 2014; Nazer et al., 2021). However, these factors were not subsequently confirmed in multivariate analyses.

## Pathophysiological Mechanisms of ARC for Severe Infections

**FIGURE 2:**
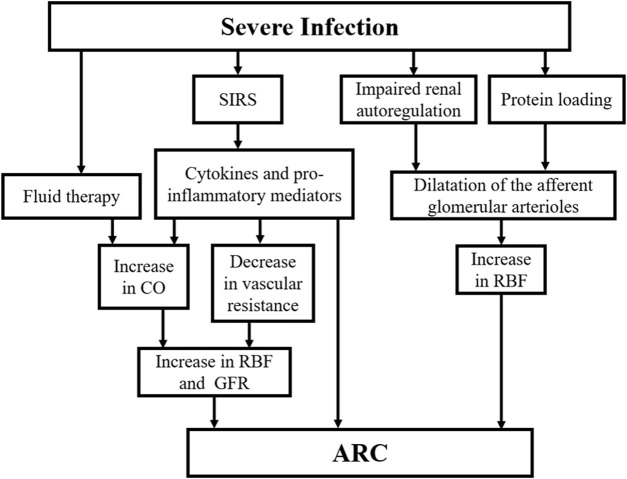
The possible pathogenesis of ARC in severe infection. ARC, augmented renal clearance; SIRS, systemic inflammatory response syndrome; RBF, renal blood flow; CO, cardiac output; GFR, glomerular filtration rate.

The mechanism of ARC for severe infections is still unclear and the ideas proposed in current studies need to be further validated. The possible pathogenesis is illustrated in Figure 2. It has been suggested that systemic inflammatory response syndrome (SIRS) caused by stressful events such as sepsis, severe trauma, burns and major surgery may be associated with ARC. When SIRS occurs, cytokines and pro-inflammatory mediators release lead to decreased vascular resistance and increased cardiac output (CO). In addition, active fluid therapy and commonly used positive inotropic drugs and vasopressors further increase CO and circulating blood volume. These factors may increase renal blood flow (RBF) and GFR (Baptista et al., 2012; Udy et al., 2012). It has recently been suggested that an increase in GFR may also reflect a direct consequence of the inflammatory process, independent of haemodynamic changes in an experimental model of endotoxemia (Beunders et al., 2020). Renal functional reserve (RFR) refers to the ability of the kidney to increase GFR in response to stress exposure. It can be used to achieve normal or supernormal renal function in the presence of increased RBF. Studies suggest that enhanced RBF through dilatation of the afferent glomerular arterioles after impaired renal autoregulation and protein loading are the main mechanisms involved in RFR mobilization (Samoni et al., 2016). SIRS in combination with greater RFR has been suggested as a possible mechanism for the development of ARC for severe infections (Campassi et al., 2014; Nazer et al., 2021).

For CNS infections, inflammatory cascade caused by cytokines released by CNS innate immune cells can lead to brain damage (Farmen et al., 2021). It has been claimed that better cerebral autoregulation ability is assessed by cerebrovascular reaction pressure index (PRx) which was defined as the correlation between arterial pressure (ABp) and intracranial pressure (ICP) may be directly related to increased renal clearance in TBI patients (Barletta et al., 2017). The mechanism behind this phenomenon in these patients may be explained by the parallelism of autoregulation to blood pressure fluctuations between vascular beds of the brain and kidney (Khalid et al., 2019). In addition, brain damage can lead to a systemic inflammatory response that subsequently causes changes in blood flow to the kidneys (Pesonen et al., 2021). Another observational study (Udy et al., 2017a) found that the increase in CrCl in patients with TBI paralleled the change in CO and a significant increase in atrial natriuretic peptide (ANP) concentrations. Whether brain-renal axis and neuroendocrine factors are also involved in ARC for CNS infections needs to be reflected and further studied. We speculate that the inflammatory state, surgical stress, and brain injury together contribute to the development of ARC in this population, which may be similar to sepsis or TBI.

## Effects of ARC on Vancomycin Pharmacokinetics/Pharmacodynamics for Severe Peripheral Infections

The “severe peripheral infections” mentioned in this paper include but are not limited to bacteremia, sepsis, septic shock, infective endocarditis, and pneumonia. However, since the patient populations in the literature we reviewed were all suspected or confirmed bacteremia, sepsis, septic shock, or CNS infections caused by Gram-positive bacteria, this concept corresponds only to bloodstream infections described above.

Due to the considerable variability in the upper range of the area under the concentration curve (AUC) associated with a given trough concentration (Cmin) (15–20 mg/L), for severe methicillin-resistant *staphylococcus aureus* (MRSA) infections, assuming a minimum inhibitory concentration (MIC) of 1 mg/L, the latest guidelines recommend an AUC of 400–600 mg ⋅ h/L as the PD target. 90% of vancomycin was cleared by the kidney, and the clearance was directly related to CrCl (Rybak et al., 2020). Despite the high incidence of ARC in severe infective patients, few studies have discussed the effect of ARC on vancomycin PK. Data are lacking to guide rational dosing in ARC patients. This is an important issue that clinicians should be aware of, as failure to meet the PD target may be associated with the eventual acquisition of resistance to antimicrobial agents (Cucci et al., 2020). Additionally, it has been established that MIC values >1 mg/L and ARC increase the risk of subexposure (Helset et al., 2020), which may have a negative effect on prognosis. Some studies demonstrated that vancomycin subexposure was an independent predictor of 30-days mortality (OR 6.83; *p* = 0.01) (Jumah et al., 2018), was significantly associated with in-hospital mortality (OR 2.1; *p* = 0.003) (Spadaro et al., 2015). For MRSA bacteremia, multivariate analyses showed that lower initial AUC/MIC was a significant risk factor for treatment failure (Song et al., 2015; Johnston et al., 2021). Seeking higher-than-target AUC/MIC may improve treatment outcomes.

Current studies have consistently concluded that CL_V_ was higher in ARC patients than in non-ARC patients. Besides, ARC patients were at high risk of subexposure, and conventional doses were not sufficient for them. Observational retrospective studies found that the ARC groups exhibited higher CL_V_ than the non-ARC groups in ICU (Hirai et al., 2016; He et al., 2020). When treated with equivalent common daily doses of vancomycin, target AUC was not achieved in all patients and was significantly lower in the ARC group (232.9 mg ⋅ h/L) than in the non-ARC group (316.2 mg ⋅ h/L) (*p* < 0.05) (He et al., 2020). Despite receiving higher doses of vancomycin, the AUC attainment rate was only 38.1% in the ARC group, which was lower than that of non-ARC patients (52.1%) (*p* < 0.0001) (Hirai et al., 2016). Moreover, CrCl was significantly negatively correlated with vancomycin concentrations on the first day of treatment (rS = −0.57, *p* < 0.001) (Baptista et al., 2012) and lower Cmin (r = 0.53, *p* ≤ 0.001) (Bakke et al., 2017). For patients with increased CrCl, Cmin could not reach the therapeutic level within 3 days even clinicians increased the dose and shortened the dosing interval (Bakke et al., 2017). A routine intermittent dosing regimen of vancomycin was significantly associated with a low Cmin attainment rate in ARC patients (Villanueva et al., 2019).

Actually, for critically ill patients, the distribution of intravenous (IV) vancomycin in systemic circulation was generally described by a one- or two-compartment model with CL_V_ described by CrCl as a significant PK parameter. CL_V_ varies with CrCl in these populations and has been reported to range from 3.97 to 7.23 L/h with high inter-individual variability (Roberts et al., 2011; Heffernan et al., 2019; Vu et al., 2019; Pongchaidecha et al., 2020). The CL_V_ observed in the study (CL_V_ = 7.23 L/h) from Heffernan et al. (2019) was higher than other studies mainly because the subjects in their study had better kidney function (median value of CrCl 107.0 ml/min). For population PK analyses of ICU patients with ARC, retrospective observational PK studies conducted by He et al. (2020) and Chu et al. (2020) reported that the ARC groups exhibited higher CL_V_, typical values were 9.7 L/h and 8.52 L/h, respectively, which were higher than those reported in other literature. According to these PK models and Monte Carlo Simulations, larger doses than the common doses recommended were required for optimal serum exposure for severe peripheral infections, especially in patients with ARC.

## Effects of Patient-Specific Factors on Vancomycin PK/PD for Severe Peripheral Infections

Population PK models of IV vancomycin have been indicated that the volume of distribution (Vd) was also an important PK parameter described by total body weight (TBW) (Roberts et al., 2011), or positively correlated with age and body weight (BW) (Pongchaidecha et al., 2020) in critically ill patients. Chu et al. (2020) retrospectively collected vancomycin concentrations samples from ARC patients with suspected or confirmed Gram-positive bacterial infections for a population PK analysis. A typical value for Vd was 155.4 L/h, which was 1.3–3.6 times higher than those reported in other literature (Roberts et al., 2011; Heffernan et al., 2019; Vu et al., 2019; Pongchaidecha et al., 2020). These may be due to increased blood flow to major organs caused by hyperdynamic state, high volume loading and increased CO. On the one hand, higher Vd levels impact serum concentrations. On the other hand, it suggests that vancomycin can be widely distributed throughout the body, which may be beneficial in treating infections in places where the drug cannot easily reach. Furthermore, Medellín-Garibay et al. (2016) proposed a two-compartment model from critically ill trauma patients receiving IV vancomycin. CrCl was associated with CL_V_ (0.49 ± 0.04 L/h) and was the most influential covariate on CL_V_. However, the use of furosemide reduced CrCl (0.34 ± 0.05 L/h). Based on the final model, they provide a new dosing regimen that considers CrCl and concomitant administration of furosemide for these patients.

In addition, some studies support that the duration and degree of the systemic inflammatory response of sepsis were also the factors to predict the plasma concentration of vancomycin. Chuma et al., 2016) found that although there were no differences in CrCl and Sequential Organ Failure Assessment (SOFA), patients with a duration of SIRS <2 days had a higher CL_V_ than those with a duration of >6 days. They hypothesized that increased capillary leakage and extravasation of blood components early in sepsis might lead to lower vancomycin concentrations. Conversely, prolonged SIRS-induced multiple organ dysfunction syndromes (MODS) may increase vancomycin levels. Zaric et al. (2018) concluded that when sepsis occurs, when renal function is not affected by infectious complications, increased CO and hyperdynamic circulation increase renal perfusion, raising GFR and allowing more drug molecules to enter the tubular lumen, where hydrophilic antibiotics cannot be reabsorbed and are passed through urinary excretion. The increase in sepsis-related renal perfusion does not lead to a sufficiently large increase in GFR in patients with renal failure with limited RFR compared to patients with ARC. Shimamoto et al. (2013) found that except for patients with renal insufficiency, CL_V_ in SIRS patients was significantly higher than in patients without SIRS. Multivariate analysis showed that CL_V_ was highly positively correlated with CrCl and SIRS scores, and negatively correlated with age, even when clearance increased. For young sepsis patients without any renal complications, increased blood flow to the kidneys may result in increased CL_V_ and lower vancomycin concentrations due to the development of high dynamic cardiovascular status and vasodilation. Therefore, larger doses of vancomycin may be considered for younger patients with shorter duration of SIRS and higher SIRS scores.

## Effects of ARC on Vancomycin PK/PD for Central Nervous System Infections

Kim et al. (2016) found infective patients after neurosurgery had significantly higher CrCl, higher CL_V_, and lower Cmin than non-neurosurgical patients. Neurosurgery and CrCl were the most important covariates explaining this higher CL_V_. Lin et al. (2016) established a one-compartment model to describe the PK process of IV vancomycin in BM patients after craniotomy. CrCl is an important covariate with a non-linear relationship with CL_V_, which significantly affects concentration. When the CrCl was 130 ml/min, the estimated CL_V_ was 9.37 L/h, which was relatively high, suggesting that ARC patients need to increase the dose. Chen et al. (2020) reported that for infective patients after neurosurgery treated with vancomycin, Cmin was lower in the ARC group compared to the normal renal function group despite receiving a higher dose (6.45 mg/L vs. 10.72 mg/L; *p* < 0.001). The rate of achieving target Cmin was 41.03% in the normal renal function group compared to only 19.23% in the ARC group. These findings indicate that infective patients after neurosurgery are at significantly higher risk of ARC and subtherapeutic serum concentrations than other patients. ARC screening combined with therapeutic drug concentration monitoring (TDM) should be performed routinely. However, for CNS infections, the difficulty in achieving therapeutic exposure in CSF is that both ARC and patients-specific factors are important elements affecting vancomycin PK/PD.

## Effects of Patients-Specific Factors on Vancomycin PK/PD for CNS Infections

Effective exposure targets at the site of infection such as CSF and the protocol required to achieve the target are difficult to determine. These cannot simply be extrapolated from the drug exposure indices in serum due to the complexity of factors affecting drug distribution and elimination in CSF. Due to its hydrophilicity, high molecular weight, and affinity for plasma proteins, vancomycin is normally poorly permeable to the blood-brain barrier (BBB) or blood-cerebrospinal fluid barrier (BCB) (Kumta et al., 2018). However, in pathological conditions such as meningitis or craniotomy which could damage the BBB or BCB, vancomycin increases permeability to CSF through the paracellular pathways, which varies greatly between individuals (Beach et al., 2017; Tiede et al., 2021). Therefore, the severity of meningeal inflammation and the degree of BBB or BCB destruction affect the concentration of CSF.

Li et al. (2015) first proposed a three-compartment model (central compartment, peripheral compartment, and CSF compartment) to describe the PK process of IV vancomycin in patients with external ventricular drainage (EVD)-associated ventriculitis. The CSF was considered a separate compartment connected to the central compartment in a first-order distribution (Q_CSF_) due to a significant time delay between CSF and plasma concentration observed after IV vancomycin. Covariate analysis showed that serum concentration and CSF albumin, which reflects the disruption degree of BBB were the factors affecting CSF concentration. Their subsequent study (Li et al., 2016) additionally identified BW as a covariate that affected the central compartment volume (V_C_) to optimize the previous model. CSF albumin level was still a covariate affecting Q_CSF_. When IV combined with intraventricular (IVT) vancomycin, the impaired BBB and BCB caused by neurosurgery made it easily permeate the CSF. Considering the drainage of the CSF by the tube also led to drug exclusion, they further proposed a three-compartment model with two elimination pathways (elimination *via* central compartment and CSF compartment) (Li et al., 2017). The process of elimination *via* the central compartment was influenced by CrCl. The drug loss from CSF can be described by the clearance of the CSF compartment (CL_CSF_), which was related to the average daily drainage amount (DA) and the elapsed time (ET) after IVT injection. Similarly, a recent PK study for patients with EVD-associated ventriculitis conducted by Jalusic et al. (2021) built a three-compartment model with two elimination pathways. They found CSF lactate levels as a substitute for inflammatory processes may be associated with increased CSF vancomycin concentration.

Blassmann et al. (2019) used the constructed three-compartment model to simulate the dosing of EVD-related ventriculitis in ICU patients. Only 5.6 and 0% of patients in the 1000 mg q12h group had CSF concentrations greater than 1 and 2 mg/L, respectively. However, with continuous infusion (CI) of 6,000 mg/d, 96.8 and 25.6% patients had CSF concentrations exceeding 1 mg/L and 2 mg/L, respectively. Larger doses may help vancomycin concentrations meet or exceed MIC in CSF.

## Optimization of Vancomycin Delivery for Severe Infective Patients With ARC

Severe infective patients have significant inter-individual variations in the distribution and elimination of hydrophilic antibiotics (Landersdorfer and Nation, 2021). Furthermore, vancomycin exposure was heavily influenced by ARC and patient-specific factors. For severe infections, previous studies have demonstrated that the standard dosing regimen was insufficient to meet PD targets (Blassmann et al., 2019; Heffernan et al., 2019; He et al., 2020). All the ICU patients who received the common dose demonstrated lower AUC than the target level of 400 mg ⋅ h/L, which showed a lower trend in the ARC group (He et al., 2020). In the 1,000 mg q12h group, almost no patients had CSF concentrations of vancomycin exceeding MIC (Blassmann et al., 2019). One-size-fits-all administration may lead to inadequate exposure, thereby increasing the risk of treatment failure and bacterial resistance. Optimizing antibiotic therapy is essential to increase targeted exposure ratios and improve clinical outcomes (Landersdorfer and Nation, 2021). Supplementary Table S1 summarizes the PK/PD literature and recommended doses of vancomycin in severe infective patients with ARC.

### Loading Dose and Continuous Infusion

Hodiamont CJ et al. evaluated the effect of 25 mg/kg vancomycin loading dose on the first 24-h PK/PD target attainment (AUC) in critically ill patients and whether it increased the risk of acute kidney injury (AKI). They observed that a 25 mg/kg loading dose of vancomycin significantly increased the rate of AUC ≥400 mg ⋅ h/L without increasing the risk of AKI (Hodiamont et al., 2021).

For severe peripheral infections, recent PK studies showed a loading dose (25–30 mg/kg) allows for rapid and effective target drug exposure (serum concentration of 20–30 mg/L) (Vu et al., 2019; Pongchaidecha et al., 2020). Monte Carlo simulations from a PK study conducted by Roberts JA et al. suggested that even higher loading doses up to 35 mg/kg were required in a group of critically ill patients with relatively better renal function (Roberts et al., 2011). The optimal maintenance dose for ARC patients depends on their CrCl. To maintain adequate exposure, 3,500 mg/d (CrCl 130 ml/min-180 ml/min) or even 4,500 mg/d (CrCl >181 ml/min) were required (Vu et al., 2019). Baptista et al. (2014), Spadaro et al. (2015) created a dosing nomogram of CI based on 8-h CrCl from 79 critically ill patients treated with vancomycin and subsequently validated its effectiveness in 25 sepsis patients. 84% of patients, including all ARC patients, achieved the target concentration of 20–30 mg/L on the first day of treatment, with negligible side effects. Patients with a CrCl of 150 ml/min required a CI of at least 3.3 g/d, while patients with a CrCl of 350 ml/min required even 5.8 g/d. During the early stages of treatment, CI based on the dosing nomogram significantly increased plasma concentrations, particularly in patients with ARC. However, many nomograms have the drawback of not being designed with PK/PD targets in critically ill patients and relying on the experience of clinicians for dose adjustment.

For CNS infection, Blassmann et al. (2019) conducted dosing simulations in patients with ventriculitis and showed that 96.8 and 25.6% in the 6,000 mg CI group had CSF concentrations exceeding 1 and 2 mg/L, respectively.

Loading dose and CI can not only regulate serum concentration and prevent gradual accumulation of vancomycin but also keep serum concentration within the therapeutic range and seem to reduce renal toxicity (Spadaro et al., 2015). Furthermore, a single concentration measured during CI allows simple AUC estimation and dose adjustment. New dosing strategies such as CI should be further evaluated in clinical trials to avoid subexposure in serum and CSF.

### Dosing Regimens Based on Population PK Models

Due to the substantial inter-individual variability in PK characteristics in critically ill patients, the personalized dosage is required to achieve therapeutic exposure in these patients (Landersdorfer and Nation, 2021). A method that predicts population PK/PD in advance without obtaining any concentrations may be needed to guide initial precise dosing. Population PK modeling should ideally be based on richly sampled vancomycin data and data extraction on important covariates associated with PK. The method estimates individual PK values using the population PK parameter equation to determine the dose, infusion time, and interval required to maintain an effective exposure (Kourogi et al., 2017). The covariates and population estimates of parameters identified by the population PK model may differ for different ICU populations due to the diversity and complexity of clinical characteristics and treatments of critically ill patients and ethnic heterogeneity (Bae et al., 2019). Different dosing recommendations can be obtained using population PK models with different covariates included (Supplementary Table S1). Numerous models represent the PK characteristics of various populations in systemic circulation. One- or two-compartment models that represent the characteristics of the target population and have been validated in the literature with reasonable predictive performance should be selected. Six published models were evaluated in two independent data sets (Guo et al., 2019). However, the model of Roberts et al. (2011) was the only model that met the validity standard and could accurately predict the concentration-time data of ICU patients in two hospitals, with the mean and median of prediction error (PE) lower than 20%.

To avoid insufficient intracranial concentration caused by using plasma TDM alone as a substitute for CSF concentration monitoring, CSF TDM is required (Jalusic et al., 2021). Simultaneous monitoring of plasma and CSF concentrations, as well as the development of an appropriate population PK model, can aid in the prediction of serum and CSF concentrations, allowing for the quantification of individual dosing for CNS infections. However, few studies have explored the PK characteristics of vancomycin in CSF (Supplementary Table S1).

Li et al. (2015) established a three-compartment model in patients with EVD-associated ventriculitis in which CSF albumin was identified as a covariate. The concentration of vancomycin in the CSF was not only affected by serum levels, but also by CSF albumin, which reflects the degree of BBB damage. Their subsequent PK study improved the previous model by adding BW as a covariate to guide the dosing schedule, which was stratified according to CSF albumin level and BW (Li et al., 2016). BW mainly affected loading dose. When BW was similar, the lower the CSF albumin (indicating less BBB damage), the higher the loading and maintenance doses required. Considerable maintenance doses (>12 g CI for 3 days) were required in patients with CSF albumin <100 mg/dl, therefore, IV is not recommended due to potential nephrotoxicity and ototoxicity (Bruniera et al., 2015). IVT or IV combined with IVT injection may be more appropriate for these patients. However, in this case, the PK characteristics of plasma and CSF are poorly understood. A recent PK study conducted by Li X et al., which considered the possible effect of CrCl on central compartment clearance, reported a three-compartment model with two elimination pathways (Li et al., 2017) to describe the PK process of vancomycin in plasma and CSF of patients with EVD-associated ventriculitis. The model showed that DA significantly affected CSF concentration, and CrCl was an important factor affecting plasma concentration. Patients were stratified according to DA and CrCl. Simulations showed that the amount of IVT injection required to achieve the target CSF exposure increased with the rise of daily DA. For patients with CrCl 150–200 ml/min, 1430 mg q12h IV injection was required and local injection was up to 70 mg q12h when the daily flow was 300–400 ml. Compared with their previous two models that only administered IV vancomycin (Li et al., 2015; Li et al., 2016), the main source of CSF drug in this study was IVT injection. The effect of damaged BBB on CSF concentration was limited, so they did not consider CSF albumin in the final model. It is important to note that the CSF outflow rate is not static, and BBB repair reduces CSF outflow rate. Thus, CL_CSF_ is a variable parameter that decreases with BBB recovery. Furthermore, simulations based on a three-compartment model established by Jalusic et al. (2021) from a recent study of EVD-associated ventriculitis showed that Cmin in CSF raised with the increase of lactate level, representing inflammatory processes in CSF. At a lactate level of 3.3 mmol/L (median of the observed population), 1350 mg q8h IV met the therapeutic goal (CSF concentration > 1 mg/L) and the recommended dose for CI was 4 g/d.

The typical CL_V_ reported in these studies was 5.15–8.75 L/h with high inter-individual variability (Li et al., 2015; Li et al., 2016; Li et al., 2017; Jalusic et al., 2021), higher than that in patients with other types of infection in the ICU. Plasma and CSF TDM monitoring is necessary for ensuring adequate exposure. Renal function changes and patient-specific factors are important considerations when designing dosing strategies for CNS infections. Currently established population PK models contribute to the further development of stratified dosing based on CrCl and patient-specific factors. Still, large-scale prospective clinical data are lacking to validate the predictive performance of different models and the safety of recommended dosing regimens.

### Dosing Regimens Based on Population PK Models Combined With Bayesian Software

Bayesian software program embedded PK data based on rich samples as a Bayesian prior PK model, using only an individualized Cmin to optimize the PK parameters of the population estimation. Once the most likely PK parameters are estimated based on a reliable model, the PK equation can be used to determine the dosing regimen and an accurate AUC, with an average bias of only 3% (Bayard and Jelliffe, 2004; Fuchs et al., 2013; Lonsdale et al., 2013; Bruniera et al., 2015). Clark et al. (2019) found a positive correlation between vancomycin Cmin and AUC in patients with suspected or confirmed MRSA infection, but 100% of patients with Cmin >9 mg/L achieved an AUC/MIC of >400 mg ⋅ h/L. Cmin of 15–20 mg/L often points to an AUC/MIC greater than that required for efficacy optimization, leading to unnecessary dose increases and risk of toxicity. A subsequent prospective study by Neely et al. (2018) confirmed that, compared with Cmin, AUC-guided Bayesian analysis of dosing was associated with reduced renal toxicity, fewer inpatient blood collections, and shorter treatment time, without affecting efficacy. Proper use of Bayesian software can help patients achieve a higher frequency of therapeutic concentration and save TDM resources (Abulfathi et al., 2018). Therefore, AUC-guided Bayesian analytical dosing is more reliable than Cmin-guided dosing. When using a single concentration for Bayesian estimation of AUC, the most accurate predictions are obtained within 1.5–6 h after infusion, although the optimal sampling time varies between different software (Shingde et al., 2020). PK parameters obtained by Bayesian methods at different sampling frequencies were estimated without bias. High sampling frequencies did not add any value (Guo et al., 2021).

Compared to traditional one- or two-compartment PK models, Bayesian software predicts targets with greater precision and accuracy by using a single concentration obtained even if it is not steady-state, allowing critically ill patients to reach target concentrations within the first 24–48 h of treatment (Cunio et al., 2020). The inclusion of covariates that can predict pathophysiological changes over time, such as changes in renal function, or additional covariates that better characterize patients’ physiological characteristics, improves the model structure, allowing Bayesian software to easily adapt to critically ill patients with rapid physiological changes (Neely et al., 2014; Kourogi et al., 2017; Turner et al., 2018; Colin et al., 2019; Kim et al., 2019). Bayesian analysis-based dosing techniques are currently provided by a range of commercial TDM packages that use different PK models. Different models are typically used for different patient subgroups within a package, and the recommended dose depends on the PK model used. A prospective observational study (Turner et al., 2018) evaluated the accuracy and bias of five commercially available Bayesian software and two first-order PK equations for estimating AUC using rich PK data collected from critically ill patients. They found that PrecisePK™ predicted the most accurate and had the smallest bias (median 5.1%). Using two concentrations, the PK equation has similar or better accuracy and bias than Bayesian software. Although estimates vary, Bayesian software represents a substantial improvement over Cmin. It was observed that a Bayesian software DoseMe^®^ significantly increased the proportion of patients with severe diabetic foot infections achieving target exposure without increasing the risk of nephrotoxicity compared with empirical dosing based on Cmin (Vali et al., 2021). With the induction of DoseMe^®^-based pilot TDM counseling services, the proportion of patients meeting vancomycin target exposure increased significantly (Stocker et al., 2021). These findings showed that Bayesian-guided dosing outperformed clinician judgment in predicting PD targets. However, these two studies were “before-after” comparative experimental studies, which were susceptible to observer effect bias, and neither of them observed any direct benefit of Bayesian-guided dosing on clinical outcome. Prospective large randomized controlled studies are still needed to assess the direct impact of Bayesian analysis on clinical outcomes and overall cost-effectiveness.

Despite the growing number of commercial Bayesian software packages, the real-world clinical practice of this approach has so far been limited (Stocker et al., 2021). The cost of software, the training of personnel with pharmaceutical knowledge, the difficulty of integrating software into existing electronic medical information systems, and the low compliance of TDM are all obstacles to the widespread use of Bayesian software in clinical practice. Furthermore, these packages employ various PK models. This poses a problem for clinicians, who must consider the limitations of various models and may be forced to switch models when treating different populations.

### Dosing Regimens Based on First-Order PK Equation (Sawchuk-Zaske Equation)

When two concentrations are obtained within the same dosing interval, the AUC can be accurately estimated by the first-order PK equation. Unlike the Bayesian approach, the equation-based approach does not use priori PK parameters of population estimation to predict individual PK parameters. Two concentrations collected within the same dosing interval allow the concentration-time curve of the dosing interval to be represented as a simple single exponential curve, using which patient-specific PK parameters can be directly calculated. Once patient-specific PK parameters are determined, the traditional one-compartment PK equation can be used to determine the dosing schedules, peak concentration (Cmax), Cmin and AUC, known as the Sawchuk-Zaske method (Sawchuk and Zaske, 1976; Pai et al., 2014a). The formula for calculating AUC for the two samples was partly based on the original method of aminoglycosides proposed by Begg et al. (1995) and modified by Pai and Rodvold (2014). However, these previous studies were based on the assumption that these drugs were administered once a day and that if they were administered multiple times a day, the AUC would be a function of the number of dosing intervals at which the same dose was administered. As an alternative to a Bayesian software, Pai MP et al. recently demonstrated that daily AUC was determined with reasonable accuracy and low bias using a simple first-order PK formula using post-distributed Cmax (1–2 h after infusion) and Cmin (Pai et al., 2014b; Pai and Rodvold, 2014).

Compared to Bayesian analysis, the disadvantage of this approach is that it lacks the flexibility of dynamic modeling, which can predict future performance and integrate additional clinical information to determine the impact on dose adjustment. The AUC based on first-order equation estimation is a static representation of the information within a specific dosing interval. If rapid changes in renal function occur during or after the sampling period, the calculation of AUC will be inaccurate (Pai et al., 2014a). In addition, it is clinically difficult to ensure an accurate collection of Cmax and Cmin. If the wrong concentration is used in the PK equation, it will lead to an incorrect estimation of AUC. On the contrary, due to its dependence on Bayesian priors, the error level has a much smaller impact on the output of Bayesian software (Turner et al., 2018).

While the ideal way to monitor AUC is to use Bayesian software, implementing such software may require expertise and extensive training and may not be cost-effective for some institutions. Equation-based AUC is familiar to clinicians and is more easily integrated into electronic health information systems. A prospective observational study (Meng et al., 2019) demonstrated that compared with Cmin-based empirical dosing, AUC-guided dosing based on the first-order PK equation improved the achievement of treatment goals (55 vs. 73.5%, *p* = 0.0014) and did not increase nephrotoxicity (9.4 vs. 11%, *p* = 0.70). An important direction for the future may be to collect Cmin and Cmax, with computers automatically outputting AUC based on equations for bedside guidance dosing.

## Limitations

Firstly, we did not analyze the influence of different MIC values on the achievement of PD target in ARC patients, since previous studies showed great variability in MIC test methods in different laboratories, and most vancomycin MIC values among MRSA isolates were 1 mg/L or lower. What is more, waiting for MIC values may result in missing the positive effect of early adequate antimicrobial therapy on patient outcomes. According to the recommendations of IDSA, MIC = 1 mg/L was assumed, and AUC was used as PD monitoring indicator before MIC data were obtained. Secondly, most of the literature reviewed was population PK studies or observational case-control or cohort studies. The effective data of vancomycin exposure mainly stem from studies of MRSA bacteremia. Prospective, large-scale, multicenter, randomized clinical trials to guide vancomycin dose optimization are urgently needed. Finally, due to the heterogeneity of the population PK study design, our narrative review was difficult to quantitatively analyze and synthesize the optimal dosing regimen, and meta-analysis may better provide practical recommendations for clinicians on vancomycin dosing regimens.

## Challenges and Perspectives

We hope this paper will alert clinicians to pay attention to severe infective patients with increased renal clearance, as ARC is associated with subexposure to hydrophilic drugs such as vancomycin, which increases the risk of bacterial resistance and treatment failure. The efficacy of antimicrobial therapy is closely related to drug exposure. Doses higher than recommended by current guidelines and individualized treatment are necessary to improve clinical outcomes in ARC patients. TDM combined with Bayesian analysis may be the best way to optimize vancomycin administration in order to increase the rate of reaching the drug exposure target and avoid nephrotoxicity. Clinicians should be cautious in selecting Bayesian software to guide drug dosing because the models included by this software are derived from different types of patients. Due to a lack of knowledge of PK/PD modeling, clinicians may not have a good understanding of the dosing recommendations generated by population PK models. In clinical practice, antibiotic PK/PD education is the key to improve the quality of the dosing strategy. Future efforts should be directed towards developing user-friendly Bayesian software that can better summarize the clinical characteristics of patients with severe infections. In addition, there is an urgent need for multi-center, large-scale, randomized controlled trials to verify the external validity of existing Bayesian models.

## Conclusion

Due to enhanced renal clearance, increased distribution and other patient-specific factors, a significant proportion of severe infective patients treated with vancomycin are at risk of subexposure. Dramatically varied PK in critically ill patients presents a great challenge to accurately predict vancomycin PD targets. The externally verified population PK model with favorable prior prediction performance seems to solve this problem. However, heterogeneity in vancomycin population PK models stems from the different study designs and patient populations in which these models were developed, suggesting the importance of using appropriate models in specific patient populations. AUC-guided Bayesian analysis allows the use of a single concentration to further optimize individual PK parameters, helping to accurately predict the dosing regimens required to achieve the target exposure. Monitoring AUC based on PK equation to guide drug dosing is an alternative method, which is well known to clinicians. Still, it lacks dynamic predictability and self-feedback compared with Bayesian modeling. Although a growing body of PK studies have confirmed that the dosing schedules guided by Bayesian analysis are superior to the traditional Cmin-guided TDM in clinical practice, large randomized controlled studies are warranted exploring the effectiveness of Bayesian analysis in improving clinical outcomes.

## References

[B1] Abulfathi A. A. Chirehwa M. Rosenkranz B. Decloedt E. H. (2018). Evaluation of the Effectiveness of Dose Individualization to Achieve Therapeutic Vancomycin Concentrations. J. Clin. Pharmacol. 58, 1134–1139. 10.1002/jcph.1254 29746714

[B2] Bae S. H. Yim D. S. Lee H. Park A. R. Kwon J. E. Sumiko H. (2019). Application of Pharmacometrics in Pharmacotherapy: Open-Source Software for Vancomycin Therapeutic Drug Management. Pharmaceutics 11, 224. 10.3390/pharmaceutics11050224 PMC6572512 31075931

[B3] Bakke V. Sporsem H. Von der Lippe E. Nordøy I. Lao Y. Nyrerød H. C. (2017). Vancomycin Levels Are Frequently Subtherapeutic in Critically Ill Patients: a Prospective Observational Study. Acta Anaesthesiol Scand. 61, 627–635. 10.1111/aas.12897 28444760 PMC5485054

[B4] Baptista J. P. Roberts J. A. Sousa E. Freitas R. Deveza N. Pimentel J. (2014). Decreasing the Time to Achieve Therapeutic Vancomycin Concentrations in Critically Ill Patients: Developing and Testing of a Dosing Nomogram. Crit. Care 18, 654. 10.1186/s13054-014-0654-2 25475123 PMC4277659

[B5] Baptista J. P. Sousa E. Martins P. J. Pimentel J. M. (2012). Augmented Renal Clearance in Septic Patients and Implications for Vancomycin Optimisation. Int. J. Antimicrob. Agents 39, 420–423. 10.1016/j.ijantimicag.2011.12.011 22386742

[B6] Barletta J. F. Mangram A. J. Byrne M. Hollingworth A. K. Sucher J. F. Ali-Osman F. R. (2016). The Importance of Empiric Antibiotic Dosing in Critically Ill Trauma Patients: Are We Under-dosing Based on Augmented Renal Clearance and Inaccurate Renal Clearance Estimates? J. Trauma Acute Care Surg. 81, 1115–1121. 10.1097/TA.0000000000001211 27533906

[B7] Barletta J. F. Mangram A. J. Byrne M. Sucher J. F. Hollingworth A. K. Ali-Osman F. R. (2017). Identifying Augmented Renal Clearance in Trauma Patients: Validation of the Augmented Renal Clearance in Trauma Intensive Care Scoring System. J. Trauma Acute Care Surg. 82, 665–671. 10.1097/TA.0000000000001387 28129261

[B8] Bayard D. S. Jelliffe R. W. (2004). A Bayesian Approach to Tracking Patients Having Changing Pharmacokinetic Parameters. J. Pharmacokinet. Pharmacodyn 31, 75–107. 10.1023/b:jopa.0000029490.76908.0c 15346853

[B9] Beach J. E. Perrott J. Turgeon R. D. Ensom M. H. H. (2017). Penetration of Vancomycin into the Cerebrospinal Fluid: A Systematic Review. Clin. Pharmacokinet. 56, 1479–1490. 10.1007/s40262-017-0548-y 28528396

[B10] Begg E. J. Barclay M. L. Duffull S. B. (1995). A Suggested Approach to Once-Daily Aminoglycoside Dosing. Br. J. Clin. Pharmacol. 39, 605–609. 10.1111/j.1365-2125.1995.tb05719.x 7654477 PMC1365071

[B11] Beunders R. Schütz M. J. van Groenendael R. Leijte G. P. Kox M. van Eijk L. T. (2020). Endotoxemia-Induced Release of Pro-inflammatory Mediators Are Associated with Increased Glomerular Filtration Rate in Humans *In Vivo* . Front. Med. (Lausanne) 7, 559671. 10.3389/fmed.2020.559671 33251227 PMC7674961

[B12] Beunders R. van de Wijgert I. H. van den Berg M. van der Hoeven J. G. Abdo W. F. Pickkers P. (2021). Late Augmented Renal Clearance in Patients with COVID-19 in the Intensive Care Unit. A Prospective Observational Study. J. Crit. Care 6464, 7–9. 10.1016/j.jcrc.2021.02.009 PMC7938790 33721609

[B13] Bilbao-Meseguer I. Rodríguez-Gascón A. Barrasa H. Isla A. Solinís M. Á. (2018). Augmented Renal Clearance in Critically Ill Patients: A Systematic Review. Clin. Pharmacokinet. 57, 1107–1121. 10.1007/s40262-018-0636-7 29441476

[B14] Blassmann U. Hope W. Roehr A. C. Frey O. R. Vetter-Kerkhoff C. Thon N. (2019). CSF Penetration of Vancomycin in Critical Care Patients with Proven or Suspected Ventriculitis: a Prospective Observational Study. J. Antimicrob. Chemother. 74, 991–996. 10.1093/jac/dky543 30689877

[B15] Bruniera F. R. Ferreira F. M. Saviolli L. R. Bacci M. R. Feder D. da Luz Gonçalves Pedreira M. (2015). The Use of Vancomycin with its Therapeutic and Adverse Effects: a Review. Eur. Rev. Med. Pharmacol. Sci. 19, 694–700. 25753888

[B16] Campassi M. L. Gonzalez M. C. Masevicius F. D. Vazquez A. R. Moseinco M. Navarro N. C. (2014). [Augmented Renal Clearance in Critically Ill Patients: Incidence, Associated Factors and Effects on Vancomycin Treatment]. Rev. Bras Ter Intensiva 26, 13–20. 10.5935/0103-507x.20140003 24770684 PMC4031886

[B17] Carrié C. Chadefaux G. Sauvage N. de Courson H. Petit L. Nouette-Gaulain K. (2019). Increased β-Lactams Dosing Regimens Improve Clinical Outcome in Critically Ill Patients with Augmented Renal Clearance Treated for a First Episode of Hospital or Ventilator-Acquired Pneumonia: a before and after Study. Crit. Care 23, 379. 10.1186/s13054-019-2621-4 31775840 PMC6881978

[B18] Chen Y. Liu L. Zhu M. (2020). Effect of Augmented Renal Clearance on the Therapeutic Drug Monitoring of Vancomycin in Patients after Neurosurgery. J. Int. Med. Res. 48, 300060520949076. 10.1177/0300060520949076 33100081 PMC7604945

[B19] Chu Y. Luo Y. Ji S. Jiang M. Zhou B. (2020). Population Pharmacokinetics of Vancomycin in Chinese Patients with Augmented Renal Clearance. J. Infect. Public Health 13, 68–74. 10.1016/j.jiph.2019.06.016 31277936

[B20] Chuma M. Makishima M. Imai T. Tochikura N. Sakaue T. Kikuchi N. (2016). Duration of Systemic Inflammatory Response Syndrome Influences Serum Vancomycin Concentration in Patients with Sepsis. Clin. Ther. 38, 2598–2609. 10.1016/j.clinthera.2016.10.009 27836495

[B21] Clark L. Skrupky L. P. Servais R. Brummitt C. F. Dilworth T. J. (2019). Examining the Relationship between Vancomycin Area under the Concentration Time Curve and Serum Trough Levels in Adults with Presumed or Documented Staphylococcal Infections. Ther. Drug Monit. 41, 483–488. 10.1097/FTD.0000000000000622 30817704

[B22] Colin P. J. Allegaert K. Thomson A. H. Touw D. J. Dolton M. de Hoog M. (2019). Vancomycin Pharmacokinetics throughout Life: Results from a Pooled Population Analysis and Evaluation of Current Dosing Recommendations. Clin. Pharmacokinet. 58, 767–780. 10.1007/s40262-018-0727-5 30656565

[B23] Cucci M. Wooten C. Fowler M. Mallat A. Hieb N. Mullen C. (2020). Incidence and Risk Factors Associated with Multi-Drug-Resistant Pathogens in a Critically Ill Trauma Population: A Retrospective Cohort Study. Surg. Infect. (Larchmt) 21 (1), 15–22. 10.1089/sur.2019.031 31210580

[B24] Cunio C. B. Uster D. W. Carland J. E. Buscher H. Liu Z. Brett J. (2020). Towards Precision Dosing of Vancomycin in Critically Ill Patients: an Evaluation of the Predictive Performance of Pharmacometric Models in ICU Patients. Clin. Microbiol. Infect. S1198-743X, 30388–8. 10.1016/j.cmi.2020.07.005 32673799

[B25] De Waele J. J. Dumoulin A. Janssen A. Hoste E. A. (2015). Epidemiology of Augmented Renal Clearance in Mixed ICU Patients. Minerva Anestesiol 81, 1079–1085. 25697881

[B26] Dhondt L. Croubels S. De Paepe P. Goethals K. De Cock P. Devreese M. (2020). Unraveling the Contribution of Fluid Therapy to the Development of Augmented Renal Clearance in a Piglet Model. Front. Pharmacol. 11, 607101. 10.3389/fphar.2020.607101 33574754 PMC7870502

[B27] Dias C. Gaio A. R. Monteiro E. Barbosa S. Cerejo A. Donnelly J. (2015). Kidney-brain Link in Traumatic Brain Injury Patients? A Preliminary Report. Neurocrit. Care 22 (2), 192–201. 10.1007/s12028-014-0045-1 25273515

[B28] Farmen K. Tofiño-Vian M. Iovino F. (2021). Neuronal Damage and Neuroinflammation, a Bridge between Bacterial Meningitis and Neurodegenerative Diseases. Front Cel Neurosci 15, 680858. 10.3389/fncel.2021.680858 PMC8209290 34149363

[B29] Fuchs A. Csajka C. Thoma Y. Buclin T. Widmer N. (2013). Benchmarking Therapeutic Drug Monitoring Software: a Review of Available Computer Tools. Clin. Pharmacokinet. 52, 9–22. 10.1007/s40262-012-0020-y 23196713

[B30] Guo T. van Hest R. M. Fleuren L. M. Roggeveen L. F. Bosman R. J. van der Voort P. H. J. (2021). Why We Should Sample Sparsely and Aim for a Higher Target: Lessons from Model-Based Therapeutic Drug Monitoring of Vancomycin in Intensive Care Patients. Br. J. Clin. Pharmacol. 87, 1234–1242. 10.1111/bcp.14498 32715505 PMC9328201

[B31] Guo T. van Hest R. M. Roggeveen L. F. Fleuren L. M. Thoral P. J. Bosman R. J. (2019). External Evaluation of Population Pharmacokinetic Models of Vancomycin in Large Cohorts of Intensive Care Unit Patients. Antimicrob. Agents Chemother. 63, e02543–18. 10.1128/AAC.02543-18 30833424 PMC6496102

[B32] He J. Yang Z. T. Qian X. Zhao B. Mao E. Q. Chen E. Z. (2020). A Higher Dose of Vancomycin Is Needed in Critically Ill Patients with Augmented Renal Clearance. Transl Androl. Urol. 9, 2166–2171. 10.21037/tau-20-1048 33209680 PMC7658164

[B33] Heffernan A. J. Germano A. Sime F. B. Roberts J. A. Kimura E. (2019). Vancomycin Population Pharmacokinetics for Adult Patients with Sepsis or Septic Shock: Are Current Dosing Regimens Sufficient? Eur. J. Clin. Pharmacol. 75, 1219–1226. 10.1007/s00228-019-02694-1 31154476

[B34] Helset E. Nordøy I. Sporsem H. Bakke V. D. Bugge J. F. Gammelsrud K. W. (2020). Factors Increasing the Risk of Inappropriate Vancomycin Therapy in ICU Patients: A Prospective Observational Study. Acta Anaesthesiol Scand. 64, 1295–1304. 10.1111/aas.13658 32578201

[B35] Hirai K. Ishii H. Shimoshikiryo T. Shimomura T. Tsuji D. Inoue K. (2016). Augmented Renal Clearance in Patients with Febrile Neutropenia Is Associated with Increased Risk for Subtherapeutic Concentrations of Vancomycin. Ther. Drug Monit. 38, 706–710. 10.1097/FTD.0000000000000346 27681114

[B36] Hobbs A. L. Shea K. M. Roberts K. M. Daley M. J. (2015). Implications of Augmented Renal Clearance on Drug Dosing in Critically Ill Patients: A Focus on Antibiotics. Pharmacotherapy 35, 1063–1075. 10.1002/phar.1653 26598098

[B37] Hodiamont C. J. Juffermans N. P. Berends S. E. van Vessem D. J. Hakkens N. Mathôt R. A. A. (2021). Impact of a Vancomycin Loading Dose on the Achievement of Target Vancomycin Exposure in the First 24 H and on the Accompanying Risk of Nephrotoxicity in Critically Ill Patients. J. Antimicrob. Chemother. 76, 2941–2949. 10.1093/jac/dkab278 34337660 PMC8521408

[B38] Huttner A. Von Dach E. Renzoni A. Huttner B. D. Affaticati M. Pagani L. (2015). Augmented Renal Clearance, Low β-lactam Concentrations and Clinical Outcomes in the Critically Ill: an Observational Prospective Cohort Study. Int. J. Antimicrob. Agents 45, 385–392. 10.1016/j.ijantimicag.2014.12.017 25656151

[B39] Jalusic K. O. Hempel G. Arnemann P. H. Spiekermann C. Kampmeier T. G. Ertmer C. (2021). Population Pharmacokinetics of Vancomycin in Patients with External Ventricular drain-associated Ventriculitis. Br. J. Clin. Pharmacol. 87, 2502–2510. 10.1111/bcp.14657 33202067

[B40] Johnston M. M. Huang V. Hall S. T. Buckley M. S. Bikin D. Barletta J. F. (2021). Optimizing Outcomes Using Vancomycin Therapeutic Drug Monitoring in Patients with MRSA Bacteremia: Trough Concentrations or Area under the Curve? Diagn. Microbiol. Infect. Dis. 101, 115442. 10.1016/j.diagmicrobio.2021.115442 34192639

[B41] Jumah M. T. B. Vasoo S. Menon S. R. De P. P. Neely M. Teng C. B. (2018). Pharmacokinetic/Pharmacodynamic Determinants of Vancomycin Efficacy in Enterococcal Bacteremia. Antimicrob. Agents Chemother. 62, e01602–17. 10.1128/AAC.01602-17 29263057 PMC5826144

[B42] Kawano Y. Morimoto S. Izutani Y. Muranishi K. Kaneyama H. Hoshino K. (2016). Augmented Renal Clearance in Japanese Intensive Care Unit Patients: a Prospective Study. J. Intensive Care 4, 62. 10.1186/s40560-016-0187-7 27729984 PMC5048448

[B43] Khalid F. Yang G. L. McGuire J. L. Robson M. J. Foreman B. Ngwenya L. B. (2019). Autonomic Dysfunction Following Traumatic Brain Injury: Translational Insights. Neurosurg. Focus 47, E8. 10.3171/2019.8.FOCUS19517 31675718

[B44] Kidney Disease: Improving Global Outcomes (Kdigo) Ckd Work Group (2013). KDIGO 2012 Clinical Practice Guideline for the Evaluation and Management of Chronic Kidney Disease. Kidney Int. Suppl. 3, 1–150.

[B45] Kim A. J. Lee J. Y. Choi S. A. Shin W. G. (2016). Comparison of the Pharmacokinetics of Vancomycin in Neurosurgical and Non-neurosurgical Patients. Int. J. Antimicrob. Agents 48, 381–387. 10.1016/j.ijantimicag.2016.06.022 27546217

[B46] Kim D. J. Lee D. H. Ahn S. Jung J. Kiem S. Kim S. W. (2019). A New Population Pharmacokinetic Model for Vancomycin in Patients with Variable Renal Function: Therapeutic Drug Monitoring Based on Extended Covariate Model Using CKD-EPI Estimation. J. Clin. Pharm. Ther. 44, 750–759. 10.1111/jcpt.12995 31228353

[B47] Kourogi Y. Ogata K. Takamura N. Tokunaga J. Setoguchi N. Kai M. (2017). Establishment of a New Initial Dose Plan for Vancomycin Using the Generalized Linear Mixed Model. Theor. Biol. Med. Model. 14, 8. 10.1186/s12976-017-0054-9 28388921 PMC5385004

[B48] Kumta N. Roberts J. A. Lipman J. Cotta M. O. (2018). Antibiotic Distribution into Cerebrospinal Fluid: Can Dosing Safely Account for Drug and Disease Factors in the Treatment of Ventriculostomy-Associated Infections? Clin. Pharmacokinet. 57, 439–454. 10.1007/s40262-017-0588-3 28905331

[B49] Landersdorfer C. B. Nation R. L. (2021). Key Challenges in Providing Effective Antibiotic Therapy for Critically Ill Patients with Bacterial Sepsis and Septic Shock. Clin. Pharmacol. Ther. 109, 892–904. 10.1002/cpt.2203 33570163

[B50] Lautrette A. Phan T. N. Ouchchane L. Aithssain A. Tixier V. Heng A. E. (2012). High Creatinine Clearance in Critically Ill Patients with Community-Acquired Acute Infectious Meningitis. BMC Nephrol. 13, 124. 10.1186/1471-2369-13-124 23013403 PMC3502432

[B51] Li X. Sun S. Ling X. Chen K. Wang Q. Zhao Z. (2017). Plasma and Cerebrospinal Fluid Population Pharmacokinetics of Vancomycin in Postoperative Neurosurgical Patients after Combined Intravenous and Intraventricular Administration. Eur. J. Clin. Pharmacol. 73, 1599–1607. 10.1007/s00228-017-2313-4 28849406

[B52] Li X. Wu Y. Sun S. Mei S. Wang J. Wang Q. (2015). Population Pharmacokinetics of Vancomycin in Postoperative Neurosurgical Patients. J. Pharm. Sci. 104, 3960–3967. 10.1002/jps.24604 26239933

[B53] Li X. Wu Y. Sun S. Zhao Z. Wang Q. (2016). Population Pharmacokinetics of Vancomycin in Postoperative Neurosurgical Patients and the Application in Dosing Recommendation. J. Pharm. Sci. 105, 3425–3431. 10.1016/j.xphs.2016.08.012 27671237

[B54] Lin W. W. Wu W. Jiao Z. Lin R. F. Jiang C. Z. Huang P. F. (2016). Population Pharmacokinetics of Vancomycin in Adult Chinese Patients with post-craniotomy Meningitis and its Application in Individualised Dosage Regimens. Eur. J. Clin. Pharmacol. 72, 29–37. 10.1007/s00228-015-1952-6 26423622

[B55] Lonsdale D. O. Udy A. A. Roberts J. A. Lipman J. (2013). Antibacterial Therapeutic Drug Monitoring in Cerebrospinal Fluid: Difficulty in Achieving Adequate Drug Concentrations. J. Neurosurg. 118, 297–301. 10.3171/2012.10.JNS12883 23121433

[B56] Mahmoud S. H. Shen C. (2017). Augmented Renal Clearance in Critical Illness: An Important Consideration in Drug Dosing. Pharmaceutics 9, 36. 10.3390/pharmaceutics9030036 PMC5620577 28926966

[B57] May C. C. Arora S. Parli S. E. Fraser J. F. Bastin M. T. Cook A. M. (2015). Augmented Renal Clearance in Patients with Subarachnoid Hemorrhage. Neurocrit. Care 23, 374–379. 10.1007/s12028-015-0127-8 25761425 PMC12968823

[B58] Medellín-Garibay S. E. Ortiz-Martín B. Rueda-Naharro A. García B. Romano-Moreno S. Barcia E. (2016). Pharmacokinetics of Vancomycin and Dosing Recommendations for Trauma Patients. J. Antimicrob. Chemother. 71, 471–479. 10.1093/jac/dkv372 26568565

[B59] Meng L. Wong T. Huang S. Mui E. Nguyen V. Espinosa G. (2019). Conversion from Vancomycin Trough Concentration-Guided Dosing to Area under the Curve-Guided Dosing Using Two Sample Measurements in Adults: Implementation at an Academic Medical Center. Pharmacotherapy 39, 433–442. 10.1002/phar.2234 30739349

[B60] Morbitzer K. A. Jordan J. D. Dehne K. A. Durr E. A. Olm-Shipman C. M. Rhoney D. H. (2019). Enhanced Renal Clearance in Patients with Hemorrhagic Stroke. Crit. Care Med. 47, 800–808. 10.1097/CCM.0000000000003716 30870191

[B61] Mulder M. B. Eidelson S. A. Sussman M. S. Schulman C. I. Lineen E. B. Iyenger R. S. (2019). Risk Factors and Clinical Outcomes Associated with Augmented Renal Clearance in Trauma Patients. J. Surg. Res. 244, 477–483. 10.1016/j.jss.2019.06.087 31330291

[B62] Nazer L. H. AbuSara A. K. Kamal Y. (2021). Augmented Renal Clearance in Critically Ill Patients with Cancer (ARCCAN Study): A Prospective Observational Study Evaluating Prevalence and Risk Factors. Pharmacol. Res. Perspect. 9, e00747. 10.1002/prp2.747 33694316 PMC7947216

[B63] Neely M. N. Kato L. Youn G. Kraler L. Bayard D. van Guilder M. (2018). Prospective Trial on the Use of Trough Concentration versus Area under the Curve to Determine Therapeutic Vancomycin Dosing. Antimicrob. Agents Chemother. 62, e02042–17. 10.1128/AAC.02042-17 29203493 PMC5786789

[B64] Neely M. N. Youn G. Jones B. Jelliffe R. W. Drusano G. L. Rodvold K. A. (2014). Are Vancomycin Trough Concentrations Adequate for Optimal Dosing? Antimicrob. Agents Chemother. 58, 309–316. 10.1128/AAC.01653-13 24165176 PMC3910745

[B65] Nei A. M. Kashani K. B. Dierkhising R. Barreto E. F. (2020). Predictors of Augmented Renal Clearance in a Heterogeneous ICU Population as Defined by Creatinine and Cystatin C. Nephron 144, 313–320. 10.1159/000507255 32428906 PMC7371523

[B66] Pai M. P. Neely M. Rodvold K. A. Lodise T. P. (2014). Innovative Approaches to Optimizing the Delivery of Vancomycin in Individual Patients. Adv. Drug Deliv. Rev. 77, 50–57. 10.1016/j.addr.2014.05.016 24910345

[B67] Pai M. P. Rodvold K. A. (2014). Aminoglycoside Dosing in Patients by Kidney Function and Area under the Curve: the Sawchuk-Zaske Dosing Method Revisited in the Era of Obesity. Diagn. Microbiol. Infect. Dis. 78, 178–187. 10.1016/j.diagmicrobio.2013.10.011 24268292

[B68] Pai M. P. Russo A. Novelli A. Venditti M. Falcone M. (2014). Simplified Equations Using Two Concentrations to Calculate Area under the Curve for Antimicrobials with Concentration-dependent Pharmacodynamics: Daptomycin as a Motivating Example. Antimicrob. Agents Chemother. 58, 3162–3167. 10.1128/AAC.02355-14 24663017 PMC4068499

[B69] Pesonen A. Ben-Hamouda N. Schneider A. (2021). Acute Kidney Injury after Brain Injury: Does it Exist? Minerva Anestesiol 87, 823–827. 10.23736/S0375-9393.20.14991-5 33054019

[B70] Pongchaidecha M. Changpadap D. Bannalung K. Seejuntra K. Thongmee S. Unnual A. (2020). Vancomycin Area under the Curve and Pharmacokinetic Parameters during the First 24 hours of Treatment in Critically Ill Patients Using Bayesian Forecasting. Infect. Chemother. 52, 573–582. 10.3947/ic.2020.52.4.573 33263245 PMC7779987

[B71] Roberts J. A. Taccone F. S. Udy A. A. Vincent J. L. Jacobs F. Lipman J. (2011). Vancomycin Dosing in Critically Ill Patients: Robust Methods for Improved Continuous-Infusion Regimens. Antimicrob. Agents Chemother. 55, 2704–2709. 10.1128/AAC.01708-10 21402850 PMC3101407

[B72] Ruiz S. Minville V. Asehnoune K. Virtos M. Georges B. Fourcade O. (2015). Screening of Patients with Augmented Renal Clearance in ICU: Taking into Account the CKD-EPI Equation, the Age, and the Cause of Admission. Ann. Intensive Care 5, 49. 10.1186/s13613-015-0090-8 26667819 PMC4681181

[B73] Rybak M. J. Le J. Lodise T. P. Levine D. P. Bradley J. S. Liu C. (2020). Therapeutic Monitoring of Vancomycin for Serious Methicillin-Resistant *Staphylococcus aureus* Infections: A Revised Consensus Guideline and Review by the American Society of Health-System Pharmacists, the Infectious Diseases Society of America, the Pediatric Infectious Diseases Society, and the Society of Infectious Diseases Pharmacists. Clin. Infect. Dis. 71, 1361–1364. 10.1093/cid/ciaa303 32658968

[B74] Samoni S. Nalesso F. Meola M. Villa G. De Cal M. De Rosa S. (2016). Intra-Parenchymal Renal Resistive Index Variation (IRRIV) Describes Renal Functional Reserve (RFR): Pilot Study in Healthy Volunteers. Front. Physiol. 7, 286. 10.3389/fphys.2016.00286 27458386 PMC4933701

[B75] Sawchuk R. J. Zaske D. E. (1976). Pharmacokinetics of Dosing Regimens Which Utilize Multiple Intravenous Infusions: Gentamicin in Burn Patients. J. Pharmacokinet. Biopharm. 4, 183–195. 10.1007/BF01086153 950590

[B76] Shimamoto Y. Fukuda T. Tanaka K. Komori K. Sadamitsu D. (2013). Systemic Inflammatory Response Syndrome Criteria and Vancomycin Dose Requirement in Patients with Sepsis. Intensive Care Med. 39, 1247–1252. 10.1007/s00134-013-2909-9 23604132

[B77] Shingde R. V. Reuter S. E. Graham G. G. Carland J. E. Williams K. M. Day R. O. (2020). Assessing the Accuracy of Two Bayesian Forecasting Programs in Estimating Vancomycin Drug Exposure. J. Antimicrob. Chemother. 75, 3293–3302. 10.1093/jac/dkaa320 32790842

[B78] Song K. H. Kim H. B. Kim H. S. Lee M. J. Jung Y. Kim G. (2015). Impact of Area under the Concentration-Time Curve to Minimum Inhibitory Concentration Ratio on Vancomycin Treatment Outcomes in Methicillin-Resistant *Staphylococcus aureus* Bacteraemia. Int. J. Antimicrob. Agents 46, 689–695. 10.1016/j.ijantimicag.2015.09.010 26555059

[B79] Spadaro S. Berselli A. Fogagnolo A. Capuzzo M. Ragazzi R. Marangoni E. (2015). Evaluation of a Protocol for Vancomycin Administration in Critically Patients with and without Kidney Dysfunction. BMC Anesthesiol 15, 95. 10.1186/s12871-015-0065-1 26116239 PMC4483208

[B80] Stocker S. L. Carland J. E. Reuter S. E. Stacy A. E. Schaffer A. L. Stefani M. (2021). Evaluation of a Pilot Vancomycin Precision Dosing Advisory Service on Target Exposure Attainment Using an Interrupted Time Series Analysis. Clin. Pharmacol. Ther. 109, 212–221. 10.1002/cpt.2113 33190285

[B81] Tiede C. Chiriac U. Dubinski D. Raimann F. J. Frey O. R. Röhr A. C. (2021). Cerebrospinal Fluid Concentrations of Meropenem and Vancomycin in Ventriculitis Patients Obtained by TDM-Guided Continuous Infusion. Antibiotics (Basel) 10, 1421. 10.3390/antibiotics10111421 34827359 PMC8614961

[B82] Tsai D. Udy A. A. Stewart P. C. Gourley S. Morick N. M. Lipman J. (2018). Prevalence of Augmented Renal Clearance and Performance of Glomerular Filtration Estimates in Indigenous Australian Patients Requiring Intensive Care Admission. Anaesth. Intensive Care 46, 42–50. 10.1177/0310057X1804600107 29361255

[B83] Turner R. B. Kojiro K. Shephard E. A. Won R. Chang E. Chan D. (2018). Review and Validation of Bayesian Dose-Optimizing Software and Equations for Calculation of the Vancomycin Area under the Curve in Critically Ill Patients. Pharmacotherapy 38, 1174–1183. 10.1002/phar.2191 30362592

[B84] Udy A. Boots R. Senthuran S. Stuart J. Deans R. Lassig-Smith M. (2010). Augmented Creatinine Clearance in Traumatic Brain Injury. Anesth. Analg 111, 1505–1510. 10.1213/ANE.0b013e3181f7107d 21048095

[B85] Udy A. A. Baptista J. P. Lim N. L. Joynt G. M. Jarrett P. Wockner L. (2014). Augmented Renal Clearance in the ICU: Results of a Multicenter Observational Study of Renal Function in Critically Ill Patients with normal Plasma Creatinine Concentrations*. Crit. Care Med. 42, 520–527. 10.1097/CCM.0000000000000029 24201175

[B86] Udy A. A. Dulhunty J. M. Roberts J. A. Davis J. S. Webb S. A. R. Bellomo R. (2017). Association between Augmented Renal Clearance and Clinical Outcomes in Patients Receiving β-lactam Antibiotic Therapy by Continuous or Intermittent Infusion: a Nested Cohort Study of the BLING-II Randomised, Placebo-Controlled, Clinical Trial. Int. J. Antimicrob. Agents 49, 624–630. 10.1016/j.ijantimicag.2016.12.022 28286115

[B87] Udy A. A. Jarrett P. Lassig-Smith M. Stuart J. Starr T. Dunlop R. (2017). Augmented Renal Clearance in Traumatic Brain Injury: A Single-Center Observational Study of Atrial Natriuretic Peptide, Cardiac Output, and Creatinine Clearance. J. Neurotrauma 34, 137–144. 10.1089/neu.2015.4328 27302851

[B88] Udy A. A. Putt M. T. Shanmugathasan S. Roberts J. A. Lipman J. (2010). Augmented Renal Clearance in the Intensive Care Unit: an Illustrative Case Series. Int. J. Antimicrob. Agents 35, 606–608. 10.1016/j.ijantimicag.2010.02.013 20307958

[B89] Udy A. A. Roberts J. A. Boots R. J. Paterson D. L. Lipman J. (2010). Augmented Renal Clearance: Implications for Antibacterial Dosing in the Critically Ill. Clin. Pharmacokinet. 49, 1–16. 10.2165/11318140-000000000-00000 20000886

[B90] Udy A. A. Roberts J. A. Lipman J. Blot S. (2018). The Effects of Major Burn Related Pathophysiological Changes on the Pharmacokinetics and Pharmacodynamics of Drug Use: An Appraisal Utilizing Antibiotics. Adv. Drug Deliv. Rev. 123, 65–74. 10.1016/j.addr.2017.09.019 28964882

[B91] Udy A. A. Roberts J. A. Shorr A. F. Boots R. J. Lipman J. (2013). Augmented Renal Clearance in Septic and Traumatized Patients with normal Plasma Creatinine Concentrations: Identifying At-Risk Patients. Crit. Care 17, R35. 10.1186/cc12544 23448570 PMC4056783

[B92] Udy A. A. Varghese J. M. Altukroni M. Briscoe S. McWhinney B. C. Ungerer J. P. (2012). Subtherapeutic Initial β-lactam Concentrations in Select Critically Ill Patients: Association between Augmented Renal Clearance and Low Trough Drug Concentrations. Chest 142, 30–39. 10.1378/chest.11-1671 22194591

[B93] Vali L. Jenkins D. R. Vaja R. Mulla H. (2021). Personalised Dosing of Vancomycin: A Prospective and Retrospective Comparative Quasi-Experimental Study. Br. J. Clin. Pharmacol. 87, 506–515. 10.1111/bcp.14411 32495366

[B94] Villanueva R. D. Talledo O. Neely S. White B. Celii A. Cross A. (2019). Vancomycin Dosing in Critically Ill Trauma Patients: The VANCTIC Study. J. Trauma Acute Care Surg. 87, 1164–1171. 10.1097/TA.0000000000002492 31464871

[B95] Vu D. H. Nguyen D. A. Delattre I. K. Ho T. T. Do H. G. Pham H. N. (2019). Determination of Optimal Loading and Maintenance Doses for Continuous Infusion of Vancomycin in Critically Ill Patients: Population Pharmacokinetic Modelling and Simulations for Improved Dosing Schemes. Int. J. Antimicrob. Agents 54, 702–708. 10.1016/j.ijantimicag.2019.09.018 31600554

[B96] Zaric R. Z. Milovanovic J. Rosic N. Milovanovic D. Zecevic D. R. Folic M. (2018). Pharmacokinetics of Vancomycin in Patients with Different Renal Function Levels. Open Med. (Wars) 13, 512–519. 10.1515/med-2018-0068 30426090 PMC6227840

