# Supplementary material for: Augmented Renal Clearance in Severe Infections—An Important Consideration in Vancomycin Dosing: A Narrative Review
Source: Front Pharmacol. 2022 Mar 21;13:835557. doi: 10.3389/fphar.2022.835557 (PMC8979486; doi:10.3389/fphar.2022.835557)
Supplement: Supplementary file 1 [file Table1.docx]

Supplementary Table 1. Summary of PK/PD literature and recommended doses of vancomycin in severe infective patients with ARC

| Author, Study type | N | Population | BW (kg) | CrCl (mL/min)  Unless otherwise stated | PK parameters | PD target used | Dosing regimen | Suggestion basis |
| --- | --- | --- | --- | --- | --- | --- | --- | --- |
| Blassmann U et al.[32],  Prospective observational PK study | 21 | Patients with proven or suspected EVD-associated ventriculitis | 76 (range 55-105) | 121.0 (range 52.3-217.6) | CL_V_ (L/h) 6.11±2.16  V_C_ (L) 24.33±11.47  K_CP_ (h^-1^) 1.44±1.22  K_PC_ (h^-1^) 2.38±1.47  K_CB_ (h^-1^) 0.18±0.23  K_BC_ (h^-1^) 0.12±0.10  V_CSF_ (L) 828.51±203.78 | CSF concentration >1 mg/L or > 2 mg/L | 6000 mg/d CI. | Monte Carlo Simulations. |
| He J et al. [52],  Retrospective observational | ARC group: 139  Non-ARC group:  141 | ICU | ARC group:  75.2±14.5  Non-ARC group:  60.4±10.0 | ARC group:  180.8±59.3^ac^  Non-ARC group:  103.4±13.3^a^ | ARC group:  CL_V_ (L/h) 9.7±3.4^d^  Vd (L/kg) 0.99±0.13  Non-ARC group:  CL_V_ (L/h) 6.4±1.7  Vd (L/kg) 0.88±0.23 | Cmin 15 mg/L | Loading dose: 25 mg/kg;  Maintenance dose: 69 mg/kg. | All the ICU patients who received the common dose demonstrated lower AUC_24_ than the target level of 400 mg·h/L, and this level showed a lower trend in the ARC group than in the non-ARC group (232.9 mg·h/L vs. 316 mg·h/L). |
| Roberts JA et al. [55],  Retrospective observational | 206 | ICU patients with severe sepsis | 74.8±15.8 | 90.7±60.4^b^ mL/min/1.73 m^2^ | CL_V_(L/h) mean 4.58  Vd(L/kg) mean 1.53 | Serum concentration 20 mg/L at steady-state | Loading dose: 35 mg/kg;  Maintenance dose: average dose is ~46 mg/kg/d in patients with measured CrCl 130 mL/min/1.73 m^2^. | Monte Carlo Simulations. |
| Heffernan, AJ et al. [56],  Retrospective observational | 27 | ICU patients with sepsis or septic shock | 75(65.5-84.8) | 107(77.3-137.8) mL/min/1.73 m^2^ | Between 0-72 h of therapy:  CL_V_(L/h) 7.23±2.57  Vd(L) 53.36±21.07  After 72 h of therapy:  CL_V_(L/h) 5.75±2.16  Vd(L) 41.61±18.22 | FTA ≥90% | In the first 24 h of therapy:  2 g q8h (CrCl 80-140 mL/min/1.73 m^2^, MIC 1 mg/L),  2 g q6h (CrCl >140 mL/min/1.73 m^2^, MIC 1 mg/L);  Between 24-72 h of therapy:  8 g/d for susceptible *E. faecium*;  The FTA was suboptimal for doses 8 g/d for susceptible coagulase-nagative *Staphylococcal* spp. And *E. faecalis.* | Monte Carlo Simulations. |
| Pongchaidech M et al. [57],  Retrospective observational | 66 | ICU | 60.8±13.5 | 51.70±41.69^a^ | CL_V_ (L/h) 2.97±1.81  Vd (L/kg) 0.85±0.22 | AUC_24_ (mg·h/L) 400-600 or ≥521 | Loading dose: 25-30 mg/kg. | Monte Carlo Simulations. |
| Vu DH et al. [58],  Retrospective observational | 55 | ICU | 55.9±11.1 | 76.5±36.4^a^ | Final Model Estimate (RSE%)  V_C_(L/kg) 1.01(15.0)  V_P_(L/kg) 2.39(23.2)  Q(L/h) 1.92(26.6)  CL_V_(L/h) 3.63(10.8) | Serum concentration 20-30 mg/L at 24 h after a loading dose of 25 mg/kg followed by maintenance dose | Loading dose: 25-30 mg/kg;  Maintenance dose: 3500 mg/d (CrCl 130-180 mL/min), 4500 mg/d (CrCl >181 mL/min). | Monte Carlo Simulations. |
| Chu Y et al. [59],  Retrospective observational | 95 | ARC Patients with Gram-positive bacteria | 70(60-80) | 175.90(142.2-198.10)^a^ | Final Model Estimate (RSE%)  CL_V_(L/h) 8.52(6.26)  Vd(L) 155.4(10.71) | NA | NA | NA |
| Medellín-Garibay SE et al. [60],  Retrospective observational | Population group: 118  Validation group: 40 | Trauma patients | Population group: 72.0±15  Validation group: 70.9±13 | Population group: 5.43±3.1 L/h  Validation group: 4.59±2.6 L/h | Population group:  CL_V_(L/h) 0.49±0.04^f^  CL_V_(L/h) 0.34±0.05^g^  Vd(L/kg) 1.07±0.11^h^  Vd(L/kg) 0.74±0.12^i^  Vd(L) 1.07±0.11  Validation group:  CL_V_(L/h) NC  Vd(L) NC | Cmin between 15-20 mg/L at steady-state and AUC_24_/MIC ≥ 400 mg·h/L | 1500 mg q12h^f^ (CrCl > 130 mL/min);  1000 mg q12h^g^ (CrCl 111-150 mL/min);  1250 mg q12h^g^ (CrCl > 150 mL/min). | Monte Carlo Simulations. |
| Baptista JP et al. [71],  Prospective observational (Group 1 data were retrospectively collected) | Group 1: 79  Group 2: 25 | ICU | Group 1: 77(70-86)  Group 2: 75(67.5-87.5) | Group 1: 125.1±66.5 mL/min/1.73 m^2^  Group 2: 120.5±54.2 mL/min/1.73 m^2^ | Group 1:  CL_V_(L/h) 5.1±1.9  Vd (L/kg) NC  Group 2:  CL_V_(L/h) NC  Vd (L/kg) NC | Serum concentration 25 mg/L at pseudo steady-state | Loading dose: 18.8(16.7-21.4) mg/kg;  CI: 3300 mg/d (CrCl 150 mL/min), 5500 mg/d (CrCl 350 mL/min). | A dosing nomogram based on 8-hour CrCl allowed all the patients with ARC belonging to Group 2 achieve the target levels. |
| Lin WW et al. [65],  Prospective observational | Model building group: 100  Validation group: 20 | Patients with post-craniotomy meningitis | Model building group: 59.1±10.0  Validation group: 62.5±12.2 | Model building group: 104.7±43.9^a^  Validation group: 98.8±45.1^a^ | Final Model Estimate (RSE%)  CL_V_(L/h) 7.56  Vd(L) 101 | Cmin 15-20 mg/L at steady-state | NA | NA |
| Li X et al. [66],  Prospective observational | 16 | Patients with EVD after neurosurgical operation recieved IV vancomycin | 69.8±9.9 | 116.2±31.5^a^ | Final Model Estimate (RSE%)  V_C_(L) 15.16(14.75)  V_P_(L) 46.10(16.78)  V_CSF_(L) 0.14(28.58)  Q(L/h) 36.97(34.94)  Q_CSF_(L/h) 0.0060(16.72)  CL_V_(L/h) 7.98(7.31)  CL_CSF_(L/h) 0.038(17.62) | CSF concentrations ≥90% MIC (MIC_90_=2 mg/L) | After a loading dose of IV 1000 mg, a larger maintenance dose is required in patients with low CSF albumin, which resulted in sustained plasma concentrations of above 40 mg/L. Thus CI is not recommended due to potential nephrotoxicity and ototoxicity. | Monte Carlo Simulations. |
| Li X et al. [67],  Prospective observational | 20 | Patients with EVD after neurosurgical operation recieved IV vancomycin | 68.90±12.07 | NA  Serum creatinine: 64.2±13.28 (μmol/L) | Final Model Estimate (RSE%)  V_C_(L) 27.84(11.47)  V_P_(L) 19.80(17.66)  V_CSF_(L) 0.12(19.62)  Q(L/h) 17.60(30.26)  Q_CSF_(L/h) 0.0049(4.08)  CL_V_(L/h) 8.75(6.35)  CL_CSF_(L/h) 0.018(7.90) | Steady-state concentration in CSF ≥ 2 mg/L | Based on the CSF albumin level and BW.  CSF albumin 100-200 mg/dL: Loading dose: IV 1000 mg, maintenance dose: IV 12 g/3d with CI.  CSF albumin < 100 mg/dL: Combined IV and IVT.  Detailed regimen is available in the literature. | Monte Carlo Simulations. |
| Li X et al. [68],  Prospective observational | 25 | Patients with EVD after neurosurgical operation recieved IV combined with IVT of vancomycin | 69.4±11.9 | 142.8±51.7^a^ | Final Model Estimate (RSE%)  V_C_(L) 11.87(19.63)  V_P_(L) 21.53(21.92)  V_CSF_(L) 0.039(12.92)  CL_V_(L/h) 7.25(10.05)  Q_CSF_(L/h) 0.0026(17.74)  CL_CSF_(L/h) 0.21(8.29) | > 90% of CSF concentrations ≥ MIC (MIC_90_=2 mg/L) | Based on the CSF DA and CrCl.  IVT doses increased as daily DA increased.  When the CrCl is 150-200 mL/min, both IV and IVT doses increased as daily DA increased.  Detailed regimen is available in the literature. | Monte Carlo Simulations.  DA had the most important influence on the CSF concentration.  ET also affected CSF concentration.  CrCl was a significant factor influencing plasma concentration. |
| Jalusic KO et al. [69],  Retrospective observational study | 29 | Neurocritically ill patients with EVD-associated ventriculitis | 80(70-85) | 152(109-174)^a^ mL/min/1.73 m^2^ | Final Model Estimate  V_C_(L) 41.13  V_P_(L) 86.20  V_CSF_(L) 0.32  Q(L/h) 3.61  Q_CSF_(L/h) 0.0031  CL_V_(L/h) 5.15 | CSF concentration > 1 mg/L | Based on the CSF lactate level and CrCl.  1350 mg q8h with a lactate of 3.3 mmol/L followed by a CI of 4 g/d is recommended in ARC patients. | Monte Carlo Simulations.  The CL_V_ depends on CrCl.  The Q_CSF_ correlates with lactate level.  CSF-to-plasma ratios correlate significantly with markers of cerebral inflammation. |

ARC: augmented renal clearance; ICU: intensive care unit; PK: pharmacokinetics; PD: pharmacodynamics; BW: body weight; CrCl: creatinine clearance; CL_V_: vancomycin clearance; Vd: volume of distribution; AUC: area under the concentration curve; Cmin: trough concentration; MIC: minimum Inhibitory concentration; EVD: external ventricular drainage; CSF: cerebrospinal fluid; V_C_: central compartment volume; V_P_: peripheral compartment volume; V_CSF_ :CSF compartment volume; Q: the clearance between central and peripheral compartments; Q_CSF_: the first-order distribution between central and CSF compartments; CL_CSF_: the clearance of the CSF compartment; K_CP_ and K_PC_: first-order transfer constants connecting the central and peripheral compartments; K_CB_ and K_BC_: first-order transfer constants connecting the central and CSF compartments; RSE: relative standard error; CI: continuous infusion; DA: drainage amount; ET: elapsed time. NC: not calculated; NA: not available; FTA: the fractional target attainment; *E. faecium*: Enterococcus faecium; *E. faecalis*: Enterococcus faecalis; IV: intravenous; IVT: intraventricular.

^a^CrCl estimated using Cockcroft-Gault equation. ^b^CrCl calculated using urinary CrCl. ^c^Comparison of CrCl in ARC patients vs. non-ARC patients, *P*<0.001. ^d^Comparison of CL_V_ in ARC patients vs non-ARC patients, p<0.01. ^e^The FTA estimates the percentage of the susceptible bacterial population that achieve the desired PK/PD (AUC_0-24_/MIC or AUC_48-72_/MIC ≥451 h^-1^) ration for a given MIC distribution. ^f^If furosemide is used. ^g^If furosemide is not used. ^h^If the patients' age > 65 years old. ^i^If the patients' age ≤ 65 years old.
